# Coping With Governmental Restrictions: The Relationship Between Stay-at-Home Orders, Resilience, and Functional, Social, Mental, Physical, and Financial Well-Being

**DOI:** 10.3389/fpsyg.2020.577972

**Published:** 2021-01-13

**Authors:** Adriana M. Barrett, Jens Hogreve, Elisabeth C. Brüggen

**Affiliations:** ^1^Department of Marketing and Supply Chain Management, Maastricht University, Maastricht, Netherlands; ^2^Ingolstadt School of Management, Catholic University of Eichstätt-Ingolstadt, Ingolstadt, Germany; ^3^KU Research Institute for Business and Economics in Service of Humanity (BESH), Catholic University of Eichstätt-Ingolstadt, Ingolstadt, Germany; ^4^BISS – Brightlands Institute for Smart Society, Maastricht University, Heerlen, Netherlands

**Keywords:** resilience, governmental restrictions, functional well-being, financial well-being, social well-being, mental well-being, physical well-being

## Abstract

The coronavirus outbreak has led to abrupt changes in people’s daily lives as many state governments have restricted individuals’ movements in order to slow the spread of the virus. We conducted a natural experiment in the United States of America in April 2020, in which we compare responses from states with “stay-at-home orders” (3 states) and no such orders (6 states). We surveyed 458 participants (55.6% female, age range 25–64, *M*_age_ = 36.5) and examined the effects of these government-imposed restrictions on social, mental, physical, and financial well-being as well as the mediating role of resilience. Structural equation modeling reveals that resilience buffers stay-at-home orders’ potential side-effects on well-being. Specifically, individuals living in states with stay-at-home orders report lower functional well-being than individuals living in states without such orders, which negatively relates to resilience. Resilience in turn is associated with higher social, mental, physical, and financial well-being. Thus, resilience can be seen as an effective means of buffering stay-at-home orders’ potential negative effects on the components of well-being. Our results indicate the central role of resilience, which is crucial in dampening the effects of stay-at-home orders on well-being. Following our results, governments and policymakers should focus their efforts on strengthening individuals’ resilience, which is a key predictor of social, mental, financial, and physical well-being.

## Introduction

The outbreak of the coronavirus disease 2019 (COVID-19) has had a major impact on society thus far. In the United States alone, nearly 11 million people have been infected and more than 240,000 people have died because of the disease as of November 16 (WHO, 2020). To slow the spread of the virus, many state governments in the United States have issued lockdown or “stay-at-home” orders that restrain people’s movements. For example, restaurants and gyms have closed, and non-essential movements have been prohibited. Policymakers have deemed this strategy necessary because there is yet no vaccine for the virus on the market and because it is not possible to reduce the spread of the virus by isolating infected individuals (Ferguson et al., 2020).

In the United States, state and local governors—rather than the national government—have been responsible for issuing stay-at-home orders to contain COVID-19. After California issued a stay-at-home order on March 19, most states followed, but at the time of writing (May 2020), seven states did not issue any stay-at-home orders at all. These circumstances created a unique opportunity to assess how different policies within the same country affect the well-being of its residents. In pursuing this opportunity, we acknowledge these orders’ importance in protecting public health since refraining from implementing such orders may have imposed unprecedented pressure on healthcare systems (Ferguson et al., 2020). Thus far, lockdown strategies have succeeded in reducing the spread of the virus as well as its associated mortality (Medeiros de Figueiredo et al., 2020). However, it is unclear how these measures impact the different components of well-being, such as individuals’ social, mental, functional, physical, and financial well-being, and whether resilience can buffer the effect of these measures on well-being.

Specifically, we use a natural experiment (Meyer, 1995), in which we compare results from three states who had a stay-at-home order at the time of data collection with results from six states who did not have a stay-at-home order over the same time frame. As participants cannot self-select by which orders they are ruled, we can view the policy as a treatment, and compare whether respondents’ perceptions of well-being differ based on this treatment (stay-at-home order vs. no stay-at-home order). We study the effect of stay-at-home orders on functional, social, mental, physical, and financial well-being. On the one hand, one may expect a negative relationship between stay-at-home orders and all components of well-being. For example, restrictions on movement may significantly affect people’s ability to carry out their normal daily activities, which could have an impact on their functional well-being. In addition, the resulting lack of contact with friends or colleagues is likely to have an impact on people’s social well-being, and early studies confirm that mental well-being also suffers since many people feel depressed or anxious as a result of these restrictions (Wright et al., 2020). Also, the effects on financial well-being are likely to be significant as such restrictions reduce economic activity to a considerable extent. On the other hand, research on other virus outbreaks did not find a substantial effect on overall subjective well-being (Lau et al., 2008). For example, Lau et al. (2008) studied the impact of the SARS outbreak in Hong Kong and concluded that there were no overall differences in life satisfaction when comparing scores from a similar sample that was gathered 1 year before the SARS-outbreak, to the sample collected during the outbreak. They only found some minor differences in overall well-being when comparing different age groups. Thus, it is interesting to investigate whether stay-at-home orders affect distinct components of well-being or not.

Moreover, we explore whether resilience mediates the impact that government restrictions may have on well-being. Resiliency concerns the resources to cope with setbacks that individuals have (Connor and Davidson, 2003; Salignac et al., 2019), and we argue that individuals’ resilience may buffer the effect of government restrictions on well-being.

Thus, we study the impact of government restrictions on well-being by answering the following research questions:

(1) What is the effect of stay-at-home orders on the components of well-being (functional, social, mental, financial, and physical)?
(2) Does resilience as a mediator attenuate the effect of stay-at-home orders on well-being?

By answering these research questions, we make two major contributions to the literature. First, we contribute to the literature on well-being by exploring the effects of government imposed restrictions on the multiple distinct components of well-being rather than just on overall well-being (i.e., life satisfaction) (e.g., Lau et al., 2008). This provides a much more nuanced understanding of the effect of stay-at-home orders on the distinct components of well-being, social, financial, physical, mental, and functional well-being. We thereby also contribute to emerging research on the effects of the COVID-19 pandemic that started to address the effects of the COVID-19 crisis on various psychological outcomes such as mental well-being (e.g., Cai et al., 2020; Nitschke et al., 2020; Paredes et al., 2020). Assessing the effects of government restrictions on the distinct components of subjective well-being creates a detailed insight into what aspects of a person’s life may be affected. Second, we contribute by generating evidence-based insights on whether resilience can mitigate government restrictions’ potential negative side-effects on well-being. Whereas some authors highlight the importance of strengthening resilience in response to the COVID-19 outbreak, they do not directly measure it (e.g., Maben and Bridges, 2020; Stark et al., 2020). Some relevant work has studied the role of resilience and mental health in specific populations such as nurses (Labrague and Santos, 2020) and students (Paredes et al., 2020), however, to our knowledge, there is no work that studies the impact of stay-at-home orders on resilience and various components of well-being in a general population. Our work recognizes the importance of resilience and creates novel insights by studying whether resilience may be temporarily reduced in response to stay-at-home order, and whether resilience buffers negative effects of stay-at-home orders on all components of well-being. In order to prepare for future virus outbreaks or other crisis situations that necessitate stay-at-home orders, evaluating how people deal with such situations is immensely important. Thus, it is essential to understand whether resiliency can be a means to cope better with such an unusual and impactful situation.

## Literature Review and Hypotheses Development

### Well-Being

An increasing consensus suggests that well-being should be the core target of governmental policies (Forgeard et al., 2011). This means that governments should focus on improving the subjective outcomes of their citizens (happiness and satisfaction), rather than objective outcomes such as GDP, when determining policy goals. Two major theoretical approaches to well-being are dominant in the literature: the hedonic approach (subjective well-being) and the eudemonic approach (psychological well-being) (Waterman, 1993; Burns et al., 2016). The hedonic approach to well-being defines well-being as the attainment of pleasure and avoidance of pain (Waterman, 1993). Diener and Lucas (1999) define subjective well-being, which is consistent with the hedonic approach, as consisting of three elements that are often summarized under the umbrella term of happiness: (1) life satisfaction, (2) the presence of positive affect, and (3) the absence of negative affect. Research has revealed that subjective well-being is affected by a wide range of determinants, including age, gender, life events, personality, and economic factors (for a review, see Diener et al., 1999). The eudemonic approach to well-being states that well-being is distinct from happiness, and instead defines well-being in terms of self-realization and living in accordance with one’s true self (Ryan and Deci, 2001). In this stream of literature, Ryff (1989) criticizes that the subjective well-being models are limited. Ryff (1989) suggests a more extensive model of well-being as measured by psychological well-being. Psychological well-being consists of six elements that capture human actualization (i.e., self-acceptance, positive relations with others, autonomy, environmental mastery, purpose in life and personal growth).

While both approaches to well-being are of theoretical and practical importance, they have distinct types of inquiry regarding the causes, consequences, and dynamics of well-being (Ryan and Deci, 2001). However, Ryan and Deci note that hedonic and eudemonic well-being are not mutually exclusive, and that there may be some significant overlap between the constructs. Following their extensive review of both approaches, Ryan and Deci (2001) conclude that well-being might be best viewed as a multidimensional phenomenon, and that most information can be obtained by measuring a variety of aspects of well-being. In the current article, we therefore focus on the assessment of multiple distinct components of well-being.

#### Component Based Approach to Well-Being

In our approach of assessing multiple distinct components of well-being, we follow the conceptualization of Halleröd and Seldén (2013) who distinguish between five components of well-being: physical, mental, psychosocial, financial, and functional. Halleröd and Seldén’s develop their conceptualization in the context of aging since they identified these components as being particularly relevant for identifying well-being issues for the elderly. However, we argue that these dimension are also highly relevant for assessing the effects of stay-at-home orders since the restrictions are likely to influence all five components. As we will explain in more detail in our method section, we follow the conceptualization of Halleröd and Seldén (2013) but since they do not provide a validated set of measures for these constructs, we rely on validated scales by other authors to measure the distinct components of subjective well-being. In the next section, we will briefly give an overview of all components and refer to how they can be measured. We argue why it is important to understand how each distinct component is affected by stay-at-home orders.

#### Definitions and Measurement of Components of Well-Being

##### Social well-being

“Social well-being” refers to feeling content with one’s social interactions and sense of community (Halleröd and Seldén, 2013). It concerns the assessment of maintenance and quality of social relations. According to Keyes (1998), social well-being can reduce when an individual’s functioning in society is challenged. Keyes describes various challenges that could harm social well-being, including reduced social coherence and a reduced sense of actively contributing to society. Stay-at-home orders reduce social gatherings. For example, sports clubs and community activities have discontinued. In addition, inhabitants of states with stay-at-home orders are discouraged from visiting family or friends (Lee, 2020). A wide range of literature shows that quarantine measures, or other forms of isolation negatively affect social well-being. For example, social well-being was reduced by placement into quarantine following the SARS outbreak (DiGiovanni et al., 2004) and the Ebola outbreak and (Denis-Ramirez et al., 2017). Similarly, isolation due to chronic disease has been found to lead to a loss of social contact (Griffiths et al., 2004). It is unclear whether these findings are similar for the current stay-at-home order as the nature of stay-at-home orders is different than that of individual quarantine. Since isolation is carried out by a large majority of the state population, rather than only by individuals who have contracted illness or who have been in close contact with someone who was infected, the consequences on social well-being may differ. For example, an increase in online interaction has taken place following the stay-at-home orders (Roose, 2020), which may mitigate the negative impact on social well-being. In addition, crises may also bring people together, which may boost social well-being. This view is supported by work of Uchida et al. (2014) who found that after a natural disaster in the form of an earthquake, people reported more feelings of social connection. The context of a natural disaster such as an earthquake differs, however, from the context of isolation due to infectious disease, as individuals are actively discouraged to see friends or family. While social media and digital contact still enable interactions between people, we expect the reduced frequency and richness of social interactions to diminish social well-being.

##### Financial well-being

Financial well-being addresses how people assess their financial situations (Halleröd and Seldén, 2013) and consists of two dimensions, current money management stress and expected future financial well-being (Netemeyer et al., 2018). This component of well-being may be affected by objective changes in one’s financial situation, such as a loss of income, but it can also be affected by financial worries or concerns (Brüggen et al., 2017). For many people, the coronavirus outbreak and subsequent lockdown measures have led to a reduction in working hours and even to job losses and, hence, a disruption in their financial situations (Statista, 2020a). In addition, many individuals are experiencing uncertainty about how a lockdown will affect the economy or their personal financial situations (Statista, 2020b). Although a reduction in spending money within some categories (e.g., restaurant visits) may also improve some people’s financial situations, we still expect that the financial shocks and worries associated with the outbreak and stay-at-home measures negatively affect financial well-being (Brüggen et al., 2017; Haisken-DeNew et al., 2019).

##### Physical well-being

Physical well-being can be affected by chronic or temporary physical illness; individuals with chronic illness or physical pain experience lower perceived health, and this effect is well documented (Hunt et al., 1980; Shields and Shooshtari, 2001). Perceived health is not only affected by illness; psychological processes, such as stress and rumination, can also have detrimental effects on perceived physical health (Tessler and Mechanic, 1978; Farmer and Ferraro, 1997; Koopmans and Lamers, 2005). Perceptions of physical well-being may thus be affected by increased anxiety and stress—for instance, by a fear of becoming ill. This fear of infection may be especially salient in states with a stay-at-home order, as individuals’ daily lives are abruptly changed to mitigate the impact of the virus. In addition, stay-at-home orders may reduce the possibility of stress-relief. In normal circumstances, individuals can reduce stress by participating in leisure activities (Coleman and Iso-Ahola, 1993). Under stay-at-home orders, these possibilities for stress-relief are limited. As stress and rumination can have a negative effect on perceived physical well-being, we expect that physical well-being will be lower in states with a stay-at-home order. While we expect the overall effect on physical well-being to be negative, it should be noted that stay-at-home orders may also have a positive effect on physical well-being, since individuals are less exposed to sources of illness in the case of a virus outbreak.

##### Mental well-being

Halleröd and Seldén (2013) state that issues in this area of well-being may be feeling of anxiety, sadness, worry, or downheartedness. Whereas Hällerod and Seldén use the term psychosocial well-being, we continue using the term “mental well-being,” as it is more prevalently used in the field. Conceptually, there is a strong overlap between these terms, since both conceptualizations assess how individuals evaluate their thoughts and feelings (Halleröd and Seldén, 2013). Following Tennant et al. (2007), we define mental well-being as encompassing three aspects: affective-emotional aspects, cognitive-evaluative aspects, and psychological functioning. Previous research into the Chernobyl disaster’s impact on mental health (Bromet, 2012) has shown that disasters significantly reduce mental well-being. For example, individuals who were exposed to the disaster, reported higher anxiety years after the disaster had occurred (Bromet, 2012). While the nature of a pandemic outbreak and the accompanying governmental measures are not directly comparable to the consequences of a nuclear disaster, the coronavirus outbreak and other disease outbreaks—and the resulting government measures—may lead to reduced mental well-being, similar to the effect of other disasters. For example, initial research following the coronavirus outbreak in China showed that psychological distress has been a huge concern during the pandemic (Qiu et al., 2020). Recent research shows that the outbreak of COVID-19 is associated with increased worry, fear, and anxiety (Cai et al., 2020; Paredes et al., 2020).

In addition to the direct impact of the outbreak on mental well-being, government-imposed restrictions may also affect mental well-being. Research on the psychological impact of quarantine measures, show that a strict quarantine (i.e., not leaving the house for work, exercise, or essential tasks such as shopping for food) has significant adverse effects on mental well-being (Brooks et al., 2020; Hossain et al., 2020). For example, individuals who were placed in quarantine as a response to the outbreak of the SARS virus reported increased psychological distress (Hawryluck et al., 2004). The scale of the stay-at-home orders that were issued in response to the COVID-19 outbreak is much larger than for the SARS outbreak, in which isolation was carried out by small sub-groups of a population. Therefore, it remains unclear whether preventive quarantine measures have a negative impact on well-being, when they are carried out by a large majority of the population, and not only by individuals who have been in direct contact with the virus. We argue that stay-at-home orders have a negative impact on mental health since they may lead to increased anxiety, worry, or rumination. In addition, stay-at-home orders reduce the availability of counseling or therapy (Xiao, 2020). Therefore, individuals who had already been experiencing lower mental well-being may experience an additional decrease in mental well-being due to the resulting loss of (professional) support.

##### Functional well-being

Functional well-being comprises the extent to which individuals are able to carry out their desired activities (Halleröd and Seldén, 2013). We argue that, of all the components of well-being, governmental restrictions are most likely to negatively affect functional well-being. Government-imposed lockdowns urge individuals not to leave their houses unless necessary (e.g., for grocery shopping or medical treatment), thereby restricting individuals from carrying out many of their desired activities. Functional well-being is often assessed in geriatric and medical research (e.g., Stewart et al., 1989; Rockwood et al., 1998) since both (chronically) ill and elderly individuals are prone to losing their functional capabilities. Findings from this domain of research show that functional limitations (Gooding et al., 1988), restrictions to activity (Benyamini and Lomranz, 2004), and low perceived control (Ruthig and Chipperfield, 2007) have a negative impact on well-being. Whereas Halleröd and Seldén (2013) and other authors most frequently describe functional well-being in the context of aging and health, a parallel can be drawn to restrictions occurring due to stay-at-home orders. In both contexts, individuals experience a reduction in the extent to which they can freely undertake what they desire to do. Whereas the source of limitation is different (physical constraints due to old age versus government orders), we believe that the activities that are impacted, and the consequences for well-being are relatively similar. Therefore, we expect stay-at-home orders to have a negative impact on functional well-being.

In summary, we hypothesize the following regarding the effects of stay-at-home orders on well-being:

H1: Stay-at-home orders have a negative effect on all components of well-being [i.e., social(H1a), financial (H1b), physical (H1c), mental (H1d) and functional (H1e) well-being].

In addition, we expect that governmental restrictions’ negative effect on functional well-being may lead to a decline in the other components of well-being. According to Halleröd and Seldén (2013), reduced well-being in one component can create a vicious cycle of negative effects on all the components of well-being. Based on this reasoning, we explore the possibility of reduced functional well-being—which we expect to be the component of well-being that governmental restrictions most affect—leading to lower scores in all the other components of well-being. Functional limitations may affect an individual’s physical health, financial situation, social contacts, and mental health. Based on these potential effects, we hypothesize the following:

H2: By reducing functional well-being, stay-at-home orders reduce physical, mental, social, and financial well-being.

### Resilience

“Resilience” is defined as the ability to cope well with adversity, trauma, tragedy, threats, or significant sources of stress (Fletcher and Sarkar, 2013). The literature on resilience encompasses a paradigm shift as it focuses on psychosocial strengths that individuals have to cope with adversity, rather than identifying risk factors that lead to malfunctioning (Richardson, 2002). In the early stages of resilience research, many studies focused on the development of children growing up under adverse circumstances (e.g., Luthar et al., 2000; Masten, 2001). As the field has developed, it now studies how a variety of populations cope with a variety of sources of adversity. In the current study our contribution is twofold: we assess the relationship between resilience and the components of well-being, and the effect of stay-at-home orders on resilience.

#### Resilience and Well-Being

Previous research has established a positive association between resilience and subjective well-being (Tomyn and Weinberg, 2018). Resilient individuals cope better with adversity, and they are able to recover from traumatizing events (Connor, 2006). Studies have demonstrated that resilience precedes a range of subjective well-being outcomes, such as mental well-being (Wu et al., 2020), happiness (Lü et al., 2014), psychological well-being (Mayordomo et al., 2016) and life satisfaction (Mak et al., 2011; Bajaj and Pande, 2016; Tomyn and Weinberg, 2018). Based on these findings, we expect that resilience may buffer stay-at-home orders’ negative effect on well-being since resilience may lead individuals to perceive themselves as capable of dealing with such adverse circumstances. Resilience can lead to a sense of being able to overcome situations such as a loss of income or a loss of social contacts. If these beliefs are strong, stay-at-home orders’ impact on well-being may not be significant, since resilience may generate a belief that such adversity is only temporary or that such problems are not of great concern. In contrast, a lack of resilience is associated with feeling helpless, which has been shown to negatively affect well-being (Minkov, 2009). Resilience may, thus, buffer the negative effects of adversity on well-being.

#### The Effect of Stay-at-Home Orders on Resilience

While we hypothesize that resilience is a key factor in mitigating stay-at-home orders’ negative side-effects on well-being, government-imposed restrictions have the potential to reduce individuals’ coping ability. Experiencing (repeated) traumatic events may reduce resilience. For example, Kessler (1997) notes that stressful events can make individuals more vulnerable and reduce their ability to recover from additional shocks. Similarly, Rutter (1981) argues that changing circumstances can affect resilience. While previous research has shown that many individuals remain resilient after experiencing trauma (Bonanno et al., 2006, 2007), it is unclear whether the same result can be expected of government-ordered lockdowns in crisis situations that may not lead to similar traumatic experience as described in the work by Bonanno et al. (2006, 2007). Still, in the context of the COVID-19 outbreak, individuals may experience a series of several negative events. The sudden restriction of movement may lead to stress (Brooks et al., 2020). In addition, individuals may experience other stressors, such as an inability to visit loved ones, a loss of income, or significant health concerns (Brooks et al., 2020). Stay-at-home orders may, thus, lead to an accumulation of negative events that could temporarily reduce individuals’ ability to cope with adversity. It is important to note that we focus on resilience measured at a specific moment, rather than the overall trait level of resilience an individual has. Thus, we conceptualize resilience as a state of being that one arrives at having experienced challenge related to stay-at-home orders. We expect that resilience may temporarily be reduced, as individuals may not yet feel capable to cope with the adverse circumstances they are experiencing. Therefore, we hypothesize the following:

H3: Stay-at-home orders have a negative effect on resilience.

H4: Resilience has a positive effect on physical, mental, social, and financial well-being.

**FIGURE 1:**
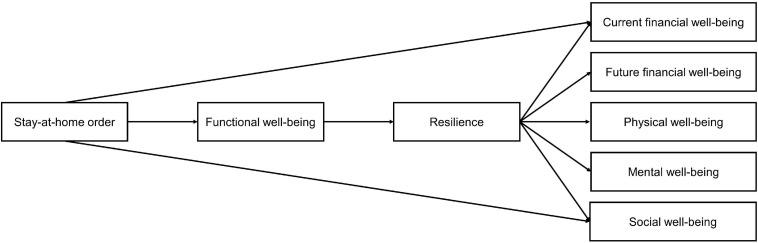
Conceptual model depicting hypothesized relationships between stay-at-home orders, resilience and components of well-being.

Figure 1 depicts the conceptual model that we have formulated based on our hypotheses.

## Materials and Methods

We analyzed the effects of government-imposed lockdown orders on well-being by comparing states that had issued stay-at-home orders with states that had not issued such orders. Our data collection took place during the COVID-19 pandemic outbreak in the spring of 2020. Participants were approached through an online panel provider (Prolific), and they were paid a monetary reward for completing our questionnaire through that provider.

### Inclusion Criteria

We sampled respondents from specific US states (stay-at-home-order states: California, Kansas, and Mississippi; no-stay-at-home-order states: Arkansas, Iowa, North Dakota, Nebraska, South Carolina, and South Dakota). Initially, 6 states were randomly selected (3 with a stay at home order, 3 without a stay at home order). However, this selection led to an oversampling of states with a stay at home order, as a large majority of our sample resided in California. Additional responses from participants with no stay-at-home order were gathered, to balance our conditions better. The data collection ran between April 6th and April 9th 2020. We coded the sampled states with regard to whether they had implemented stay-at-home orders at the time of measurement, based on a frequently updated national newswebsite (Mervosh et al., 2020). The sample states’ policies regarding stay-at-home orders did not change during our data collection. Nine participants were removed from our sample because they did not meet our state selection criterion. Eleven participants were removed because they failed an attention check at the beginning of the survey. Finally, one participant was removed because they did not fall within our predetermined age range.

### Sample

Our final sample consisted of 459 participants (55.6% female, 42.7% male, 1.7% other). We sampled individuals between 25 and 64 years of age (*M*_age_ = 36.46, *SD* = 10.44). This age range is frequently used when studying the working population (e.g., Bissonnette and van Soest, 2015), our population of interest. In this age range, most individuals will no longer be studying, and are not retired yet. The states with and without stay-at-home orders reflected some demographic differences (see Table 1).

**TABLE 1:** Differences in demographics between states with or without a stay-at-home order.

Variable	No stay-at-home order %, mean (*SD*)	Stay-at-home order %, mean (*SD*)	Test	*p*
Gender	36.9% male	48.8% male	χ^2^	0.011
Age	38.51 (10.86)	34.89 (9.86)	Manova	<0.001
Income	5.78 (3.16)	6.44 (3.46)	Manova	0.041
Amount of new Covid-19 infections per 100k inhabitants during data collection	2.91 (0.04)	3.36 (0.04)	Manova	<0.001
	**No stay-at-home order**	**Stay-at-home order**		
State	Arkansas (*N* = 46) Iowa (*N* = 73) North Dakota (*N* = 14) Nebraska (*N* = 38) South Carolina (*N* = 20) South Dakota (*N* = 8)	California (*N* = 234) Kansas (*N* = 14) Mississippi (*N* = 11)		
Starting date stay at home-order	N/A	California – March 19 Kansas – March 30 Mississippi – April 06		

*Income was measured on a 11-point scale with steps of $10,000.*

A chi-square test indicated that the proportion of males in the sample was larger for the states with stay-at-home orders [48.8% male vs. 36.9%; χ^2^ (1) = 6.43, *p* = 0.011]. A MANOVA analysis revealed differences in age, income, and number of new COVID-19 infections [*F*(3,424) = 25.85, *p* < 0.001] between states with stay-at-home orders and states without such orders. In states with stay-at-home orders, the average age (*M* = 34.89, *SD* = 9.86) was lower than in states without such orders (*M* = 38.51, *SD* = 10.86). Furthermore, household income was higher in states with stay-at-home orders (*M* = 6.44, *SD* = 3.46) than in states without such orders (*M* = 5.78, *SD* = 3.16). Finally, the number of new COVID-19 cases per 100,000 inhabitants reported in each state during our data collection period^1^ was higher in states with stay-at-home orders (*M* = 3.36, *SD* = 0.04) than in states without such orders (*M* = 2.91, *SD* = 0.04). We account for these differences in our analysis by adding them as control variables.

### Procedure

Participants first provided self-reported measures of resilience, social well-being, financial well-being, perceived health, mental well-being, and functional well-being (see Table 2 for an overview of the descriptive statistics and reliability per scale, and see Table 3 for a detailed overview of all the measures included in the survey). We randomized the order of these scales among participants in the questionnaire. The survey concluded by measuring demographic variables.

**TABLE 2:** Descriptive statistics and Pearson’s correlations for resilience and components of well-being (*N* = 459).

Scale	# items	Range	α	*M*	*SD*	1	2	3	4	5	6
(1) Resilience	10	1–5	0.91	3.53	0.74	–					
(2) Social well-being	5	1–5	0.81	2.49	0.88	0.48***			–		
(3) Future financial well-being	5	1–5	0.92	3.00	1.01	0.47***	0.43***			–	
(4) Current financial well-being	5	1–5	0.84	3.21	1.00	0.40***	0.61***	0.34***			–
(5) Physical well-being	7	1–5	0.85	3.11	0.82	0.43***	0.35***	0.36***	0.25***		–
(6) Mental well-being	14	1–5	0.93	3.12	0.75	0.70***	0.50***	0.47***	0.60***	−0.46***	
(7) Functional well-being	4	1–5	0.65	3.02	0.93	0.18***	0.06	0.08	0.08	0.23***	0.07

***p < 0.001.

**TABLE 3:** Overview of scales and items measured in survey.

Scale	# point scale	Item
**Resilience** Campbell-Sills and Stein (2007).	5 (not true at all, true nearly all the time)	1. Able to adapt to change. 2. That I can deal with whatever comes. 3. I try to see humorous side of problems. 4. That coping with stress can strengthen me. 5. I tend to bounce back after illness or hardship. 6. That I can achieve goals despite obstacles. 7. That I can stay focused under pressure. 8. I am not easily discouraged by failure. 9. I think of myself as a strong person. 10. I can handle unpleasant feelings.
**Social well-being** subscale of Evaluating the Psychometric Properties of the Mental Health Continuum-Short Form (MHC-SF) Lamers et al. (2011).	5 (never – every day)	1. That you had something important to contribute to society. 2. That you belonged to a community (like a social group, your neighborhood, your city). 3. That our society is becoming a better place for people. 4. That people are basically good. 5. That the way our society works makes sense to you.
**Financial well-being** Netemeyer et al. (2018).	5 (does not describe me at all – describes me completely)	Expected Future Financial Security. 1. I am becoming financially secure. 2. I am securing my financial future. 3. I will achieve the financial goals that I have set for myself. 4. I have saved (or will be able to save) enough money to last me to the end of my life. 5. I will be financially secure until the end of my life. Current Money Management Stress: 1. Because of my money situation, I feel I will never have the things I want in life.* 2. I am behind with my finances.* 3. My finances control my life.* 4. Whenever I feel in control of my finances, something happens that sets me back.* 5. I am unable to enjoy life because I obsess too much about money.*
**Current health perception** Ware (1976).	5 (definitely false, definitely true)	1. I feel better now than I ever have before. 2. I am somewhat ill*. 3. I am not as healthy now as I used to be*. 4. I am as healthy as anybody I know. 5. My health is excellent. 6. I have been feeling bad lately*. 7. I feel about as good now as I ever have.
**Mental well-being** Tennant et al. (2007).	5 (none of the time – all the time)	1. I’ve been feeling optimistic about the future. 2. I’ve been feeling useful. 3. I’ve been feeling relaxed. 4. I’ve been feeling interested in other people. 5. I’ve had energy to spare. 6. I’ve been dealing with problems well. 7. I’ve been thinking clearly. 8. I’ve been feeling good about myself. 9. I’ve been feeling close to other people. 10. I’ve been feeling confident. 11. I’ve been able to make up my own mind about things. 12. I’ve been feeling loved. 13. I’ve been interested in new things. 14. I’ve been feeling cheerful.
**Functional well-being** – Own Measure. Halleröd and Seldén (2013) provide a list of indicators that represent issues of functional well-being. Our scale builds on these objective indicators (e.g., not able to carry out a hobby and limited mobility) as a starting point and assesses subjective perceptions of limited mobility, and limitations in carrying out everyday activities. Further, we included two items that are in line with the current context of government restrictions, in which we ask participants to assess how restrictive they perceive these measures to be. In line with other measures of well-being, we asked respondents about their perceptions on a continuous scale.	5 (strongly disagree – strongly agree)	1. I am able to perform my daily activities as usual. 2. I feel refrained in my mobility*. 3. The shutdown in the state I live in is restrictive*. 4. I personally perceive the shutdown in the state that I live in as restrictive*.

*Reverse scored item.

### Measures

#### Social Well-Being

To assess social well-being, we used the social well-being subscale of the Mental Health Continuum-Short Form (Lamers et al., 2011). This five-item subscale assesses the extent to which people feel positive about their social lives. For example, respondents were asked to indicate how frequently they felt that they belong to a community. The psychometric qualities of this subscale are good; the authors report the subscale has adequate internal reliability and good convergent validity (Lamers et al., 2011). We included an instruction to assess the frequency of these feelings since the coronavirus outbreak (Cronbach’s alpha = 0.81).

#### Financial Well-Being

The perceived financial well-being scale developed by Netemeyer et al. (2018) measures two dimensions of financial well-being. In a series of studies, Netemeyer et al. (2018) assessed the reliability and validity of these scales and concluded that the scales are suitable for measuring individual differences in current and future financial well-being. With this subjective scale, individuals are asked to indicate on a five-point scale the extent to which 10 items describe them or do not describe them. The items are evenly distributed over the scale’s two dimensions. The first dimension is current money management stress (α = 0.84). An example of a (reverse-scored) item in this subscale is, “I am unable to enjoy life because I obsess too much about money.” The second part of the scale measures expected future financial security (α = 0.92). One of the items assessed for this dimension is, “I am securing my financial future”.

#### Physical Well-Being

We measured physical well-being using the health perception subscale of Ware’s (1976) general health perception scale. This scale assesses individuals’ subjective perceptions of their current health situations. In a range of tests, the health perception subscale shows good reliability, validity, and temporal stability (Ware, 1976). The original scale consists of nine items. However, in our survey, we removed two items that were specific to patients under a doctor’s supervision and therefore do not fit our context. The remaining seven items have an α = 0.85.

#### Mental Well-Being

We assessed mental well-being using the Warwick-Edinburgh Mental Well-being Scale (Tennant et al., 2007). The unidimensional scale consists of 14 items for which respondents are asked to indicate how often they feel a specific way. For example, individuals were asked to indicate how often they felt good about themselves. Tennant et al. (2007) report that the scale has high reliability in a general population sample. In addition, scale validation testing showed that the content and construct validity of the scale is good. We adapted the instruction text to assess how frequently people felt these feelings since the coronavirus outbreak (α = 0.93).

#### Functional Well-Being

We constructed four items to assess the extent to which stay-at-home orders have affected people’s daily functioning in life and, thus, their functional well-being. If people feel restricted, for example by instructions to reduce their movements, this implies reduced functional well-being, as people do not feel good about their functional abilities (Halleröd and Seldén, 2013). Halleröd and Seldén (2013) provide a list of indicators that represent issues of functional well-being. Our scale builds on these objective indicators (e.g., not able to carry out a hobby, limited mobility) as a starting point and assesses subjective perceptions of limited mobility, and limitations in carrying out everyday activities. Further, we included two items that are in line with the current context of government restrictions, in which we ask participants to assess how restrictive they perceive these measures to be. In line with other measures of well-being, we asked respondents about their perceptions on a 5-point scale ranging from strongly disagree to strongly agree. The items about restrictions and restrained mobility were reverse-scored, with higher scores indicating lower functional well-being. The exact wording of these items can be found in Table 3. The reliability of the scale was 0.65, which is not extremely high but passes conventional threshold levels (Taber, 2018).

#### Resilience

The survey also contained a 10-item resilience scale (Campbell-Sills and Stein, 2007). This scale is an abbreviated version of the original Connor-Davidson Resilience Scale (Connor and Davidson, 2003). This brief scale assesses an individual’s resilience in coping with adversity (Connor and Davidson, 2003; Campbell-Sills and Stein, 2007). The brief resilience-scale demonstrates excellent psychometric qualities, and is a valid and reliable measure for resilience (Campbell-Sills and Stein, 2007). An example item is, “I can deal with whatever comes.” We altered the survey’s introduction text so that it applied to the context of a pandemic by asking individuals to indicate how they had felt since the coronavirus outbreak (α = 0.91).

## Results

**FIGURE 2:**
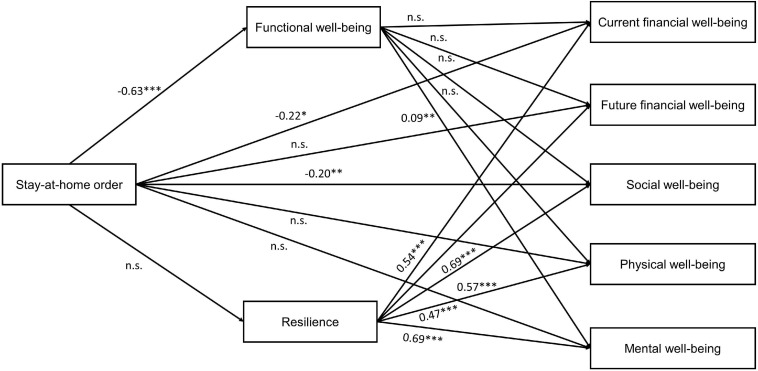
Path coefficients for hypothesized model.

We analyzed the data through structural equation modeling. Specifically, we used the Lavaan package in R (Rosseel, 2012) to test whether functional well-being and resilience mediated the relationship between stay-at-home orders and the components of well-being (see Figure 2 for an overview of all path coefficients in the model). Firstly, we test the effect of stay-at-home orders on the components of well-being. In support of hypothesis 1a, we found that social well-being was lower in states with stay-at-home orders (β = −0.200; CI_95_ [−0.347; −0.045], *p* = 0.009). Hypothesis 1b was partially supported, as we found negative effects of stay-at-home orders on current financial well-being (β = −0.222; CI_95_ [−0.403; −0.029], *p* = 0.019), but not on future financial well-being. There was no significant difference in physical well-being between states with or without stay-at-home orders, therefore hypothesis 1c is not confirmed. Similarly, hypothesis 1d was not confirmed, as there were no significant differences in mental well-being based on whether there was a stay-at-home order in place. Finally, we found that stay-at-home orders have a strong and positive effect on functional well-being (β = −0.635; CI_95_ [−0.792;.471], *p* < 0.001), supporting H1e.

Secondly, we test the effect of functional well-being on the other components of well-being. In line with hypothesis 2, we found that functional well-being has a significant relationship with mental well-being (β = 0.094; CI_95_ [0.037; 0.150], *p* = 0.001), which demonstrates that lower functional well-being is associated with lower mental well-being. However, functional well-being does not have a significant relationship with the other components of well-being: social well-being (β = −0.043; CI_95_ [−0.121; 0.035], *p* = 0.268), current financial well-being (β = −0.035; CI_95_ [−0.133; 0.065], *p* = 0.497), future financial well-being (β = −0.061; CI_95_ [−0.162; 0.045], *p* = 0.244), and physical well-being (β = −0.003; CI_95_ [−0.090; 0.083], *p* = 0.944). Thus, the results did not support H2.

Thirdly, we test whether resilience mediates the relationship between stay-at-home orders and well-being. Contrary to H3, we found that stay-at-home orders do not have a direct effect on resilience (β = −0.113; CI_95_ [−0.250; 0.025], *p* = 0.103). However, supporting H4, we did find positive effects for the relationships between resilience and social well-being (β = 0.572; CI_95_ [0.472; 0.675], *p* < 0.001), current financial well-being (β = 0.540; CI_95_ [0.425; 0.651], *p* < 0.001), future financial well-being (β = 0.690; CI_95_ [0.579; 0.804], *p* < 0.001), mental well-being (β = 0.688; CI_95_ [0.615; 0.759], *p* < 0.001), and physical well-being (β = 0.468; CI_95_ [0.375; 0.558], *p* < 0.001).

Based on these findings, we revised our model. Since we did not find a direct association between stay-at-home orders and resilience, we hypothesized that stay-at-home orders’ negative effects on resilience may only occur for individuals whose functional well-being is affected. If individuals’ day-to-day activities (and, thus, their functional well-being) are not limited despite their state having stay-at-home orders in place, we would not expect their resilience to be reduced. This expectation is in line with research by Martin (2015), who argues that when a disaster affects individuals’ daily circumstances, it reduces their resilience to cope with adversity. In addition, Martin (2015) argues that resilience may reduce when individuals feel they are not in control of their own decision-making. Since functional well-being concerns the extent to which individuals are free to conduct their daily activities as usual, without feeling restricted by stay-at-home orders, a reduced sense of functional well-being may be associated with reduced resilience. In addition, for simplicity, and since we did not find significant associations between functional well-being and most of the other components of well-being, we removed the direct paths from functional well-being to the other components of well-being from our model.

Analysis of model fit indicated that our adjusted model fit the data well (χ^2^ = 22.01, *p* = 0.001, *df* = 6; CFI = 0.986, TLI = 0.934, RMSEA = 0.076, SRMR = 0.020).

**FIGURE 3:**
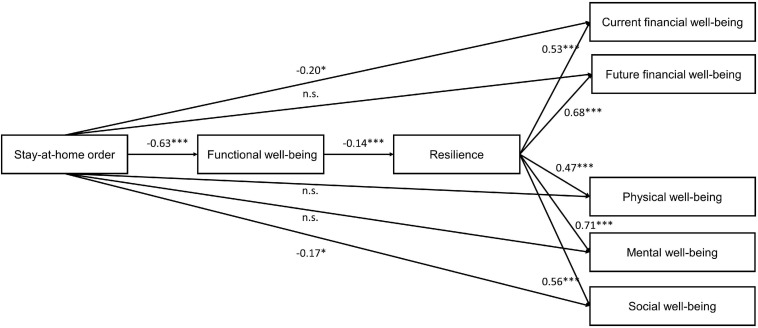
Path coefficients for revised model.

Figure 3 presents the results of our model.

When we examined stay-at-home orders’ overall effects, we found both significant direct effects and significant indirect effects. When we look at the direct effects, we find significant differences for social and current financial well-being of respondents from states with stay-at-home orders to those who were not under such an order. Respondents from states with stay-at-home orders reported lower levels of social (β = −0.174; CI_95_ [−0.317; −0.037], *p* = 0.017) and current financial well-being (β = −0.201; CI_95_ [−0.380; −0.041], *p* = 0.021), which supports H1a and H1b. The direct effects on future financial well-being (β = −0.102; CI_95_ [−0.289; 0.073], *p* = 0.273), physical (β = 0.037; CI_95_ [−0.099; 0.182], *p* = 0.600) and mental (β = 0.001; CI_95_ [−0.101; 0.098], *p* = 0.989) well-being are not significant. In line with H1e, for states with stay-at-home orders, individuals reported lower functional well-being (β = −0.635; CI_95_ [0.484; 0.792], *p* < 0.001)—which is, in turn, associated with lower resilience (β = −0.143; CI_95_ [−0.221; −0.064], *p* < 0.001). The structural equation modeling results reveal that resilience buffers stay-at-home orders’ potential side-effects on all the components of well-being.

When inspecting stay-at-home orders’ indirect effects on well-being (via functional well-being and resilience), our results show that these paths are significant for *all* the well-being components (see Table 4). These indirect effects indicate that individuals who experience reduced functional well-being because of stay-at-home orders in their states are more likely to report lower levels of resilience—which are, in turn, associated with lower financial, social, mental, and physical well-being. Our model explains between 17.1 and 48.5% of the variance in the well-being components (current financial well-being *R*^2^ = 0.171*;* physical well-being *R*^2^ = 0.180; future financial well-being *R*^2^ = 0.219; social well-being *R*^2^ = 0.242; mental well-being *R*^2^ = 0.486). For an overview of all the tested hypotheses, see Table 5.

**TABLE 4:** Path coefficients for direct and indirect effects of stay-at-home orders on resilience and well-being components.

Path	Coef.	*SE* (bootstrapped)	*P*-value	CI95
**Direct effects**				
Stay-at-home order → Social well-being	−0.174	0.073	0.017*	−0.317; −0.037
Stay-at-home order → Current financial well-being	−0.201	0.087	0.021*	−0.380; −0.041
Stay-at-home order → Future financial well-being	−0.102	0.093	0.273	−0.289; 0.073
Stay-at-home order → Physical well-being	0.037	0.071	0.600	−0.099; 0.182
Stay-at-home order → Mental well-being	0.001	0.051	0.989	−0.101; 0.098
Stay-at-home order → Functional well-being	−0.635	0.079	< 0.001***	−0.484; −0.792
Functional well-being → Resilience	0.143	0.040	< 0.001***	0.221; 0.064
**Indirect effects**				
Stay-at-home order → Functional well-being → Resilience → Social well-being	−0.051	0.017	0.003**	−0.090; −0.021
Stay-at-home order → Functional well-being → Resilience → Future financial well-being	−0.062	0.021	0.003**	−0.109; −0.026
Stay-at-home order → Functional well-being → Resilience → Current financial well-being	−0.049	0.017	0.004**	−0.089; −0.021
Stay-at-home order → Functional well-being → Resilience → Physical well-being	−0.043	0.015	0.004**	−0.077; −0.018
Stay-at-home order → Functional well-being → Resilience → Mental well-being	−0.064	0.022	0.003**	−0.111; −0.027

*p < 0.05, **p < 0.01, ***p < 0.001.

**TABLE 5:** Overview of tested hypotheses.

Hypothesis	Supported	Explanation
H1a: Stay-at-home orders have a negative effect on social well-being	Yes	We find a negative direct effect of stay-at-home orders on social well-being. In addition, we find a negative indirect effect via reduces functional well-being and reduced resilience.
H1b: Stay-at-home orders have a negative effect on financial well-being	Yes (partially)	We find a negative direct effect of stay-at-home orders on current financial well-being. We do not find a significant direct effect on future financial well-being. We find a negative indirect effect via reduces functional well-being and reduced resilience on both current as well as future financial well-being.
H1c: Stay-at-home orders have a negative effect on physical well-being	No	We do not find a significant direct effect of stay-at-home orders on physical well-being. However, we do find a negative indirect effect via reduced functional well-being and reduced resilience.
H1d: Stay-at-home orders have a negative effect on mental well-being	No	We do not find a significant direct effect of stay-at-home orders on mental well-being. However, we do find a negative indirect effect via reduced functional well-being and reduced resilience.
H1e: Stay-at-home orders have a negative effect on functional well-being	Yes	We find a negative direct effect of stay-at-home orders on functional well-being.
H2: Reduced functional well-being has a negative effect on physical, mental, social, and financial well-being	No	We do not find associations between functional well-being and most components of well-being. However, lower functional well-being is associated with lower mental well-being.
H3: Stay-at-home orders have a negative effect on resilience.	No	We do not find a direct significant relationship between stay-at-home orders and resilience. However, for those individuals whose functional well-being is reduced due to stay-at-home orders, we find a significant negative association with resilience.
H4: Resilience has a positive effect on physical, mental, social, and financial well-being	Yes	Resilience is significantly associated with all components of well-being.

### Robustness Check

As there were *a priori* differences between the states with or without stay-at-home orders, we ran a MANCOVA analysis to assess whether the effects of stay-at-home orders on well-being and resilience were robust when controlling for these *a priori* differences. We examined the effect of stay-at-home orders and gender on resilience and all components of well-being (functional, financial, physical, mental, and social). Furthermore, we included age, income and the number of new COVID-19 cases that emerged in the state during the data collection period as covariates (see Table 6). Upon inspecting the effects of stay-at-home orders on well-being, we find that the negative effects of stay at home orders on current financial well-being [*F*(1,414) = 4.35, *p* = 0.038] social well-being [*F*(1,414) = 5.34, *p* = 0.021], and functional well-being [*F*(1,414) = 40.43, *p* < 0.001] are robust when controlling for age, income and the number of new COVID-19 cases during data collection (see Table 7). Thus, these results indicate that the direct effects of stay-at-home orders on functional, social and current financial well-being hold when controlling for a set of possible confounding variables.

**TABLE 6:** Results multivariate tests of effects stay-at-home order, gender, age, income and #new covid infections per 100k inhabitants on resilience and components of well-being.

Effect	Test statistic	Value	*F*	*df*	*p*	Partial η^2^
Intercept	Wilks’ Lambda	0.610	37.293	7, 408	<0.001	0.390
Stay-at-home	Wilks’ Lambda	0.876	8.248	7, 408	<0.001	0.124
Gender	Wilks’ Lambda	0.968	1.918	7, 408	0.065	0.032
Age	Wilks’ Lambda	0.922	4.911	7, 408	<0.001	0.078
Income	Wilks’ Lambda	0.854	9.943	7, 408	<0.001	0.146
New Covid cases	Wilks’ Lambda	0.989	0.632	7, 408	0.729	0.078

**TABLE 7:** Effects of stay-at-home orders on resilience and well-being, whilst controlling for gender, age, income and #new covid infections per 100k inhabitants.

Variable	*MS*	*F*	*df*	*p*	Partial η^2^
Social well-being	4.042	5.336	1, 414	0.021	0.013
Current financial well-being	3.917	4.350	1, 414	0.038	0.010
Future financial well-being	3.514	3.394	1, 414	0.066	0.008
Physical well-being	0.111	0.165	1, 414	0.685	<0.001
Mental well-being	0.130	0.240	1, 414	0.625	0.001
Functional well-being	31.174	40.426	1, 414	<0.001	0.089
Resilience	0.231	0.435	1, 414	0.510	0.001

*MS, mean squares.*

In addition, we conducted robustness checks through structural equation modeling, including the same control variables as in the MANCOVA analysis reported above. These analyses also confirmed that our results are robust when controlling for differences in age, gender, income, and number of COVID-19 infections per state. These analyses are reported in the Supplementary Material.

## Discussion

We made observations based on naturally occurring differences during the COVID-19 pandemic to study the effect of government-imposed restrictions on resilience and on the multiple distinct components of well-being. The results of our natural experiment indicate that stay-at-home orders have a negative effect on social well-being as well as financial well-being (current money management stress). All the other components of well-being are not directly affected by stay-at-home orders. However, we found a negative indirect relationship between stay-at-home orders, functional well-being, resilience, and all the components of well-being. Our results indicate that individuals living in states with stay-at-home orders report lower functional well-being than individuals living in states without such orders, which negatively relates to resilience. Resilience, however, positively relates to social, mental, physical, and current as well as future financial well-being.

The finding that stay-at-home orders have a negative direct effect on social well-being is consistent with previous literature that demonstrates that in situations where social contacts are restricted, social well-being is reduced (DiGiovanni et al., 2004; Griffiths et al., 2004; Denis-Ramirez et al., 2017). Therefore, it is not surprising that the COVID-19 pandemic has not promoted social connectedness, contrary to other natural disasters (Uchida et al., 2014). A unique feature of stay-at-home orders is that they limit social contacts by introducing social distancing. Social distancing refers to keeping a physical distance from individuals outside one’s household, to reduce the odds of disease transmission. While virtual interaction has been possible during stay-at-home orders, the richness or frequency of these online interactions has, apparently, not compensated for the reduction in personal interactions. Our results indicate that social well-being is lower in states that have stay-at-home orders in place. The reduced frequency of physical socializing may put specific groups, such as individuals who live alone and the elderly, at risk of social isolation (Douglas et al., 2020). According to Abramson et al. (2015) social resources are of great importance for coping with disaster. Therefore, governments should be aware that stay-at-home orders may be associated with reduced social well-being.

Our finding that current financial well-being is lower in states with stay-at-home orders is noteworthy because incomes in states with stay-at-home orders were significantly higher than incomes in states without such orders. Previous research has linked higher incomes to higher financial well-being (Netemeyer et al., 2018). However, in our study, the negative relationship between stay-at-home orders and current financial well-being is robust when controlling for income. It may be that changes in one’s financial situation (e.g., the loss of a job, a reduction in income) rather than one’s absolute financial situation negatively affect financial well-being. Previous research has shown that the loss of employment is a financial stressor, that negatively affects financial satisfaction (Joo and Grable, 2004). In addition, stay-at-home orders may, possibly, have led not only to changes in people’s actual financial situations but also to a negative effect on people’s financial well-being through increased insecurity or worry about their current financial situations. Stay-at-home orders’ direct effect on financial well-being is limited to current money management stress; it does not influence expected future financial security. Thus, our results indicate that stay-at-home orders may cause stress about people’s day-to-day money management but have not directly affected people’s sense of long-term financial security.

Contrary to our hypotheses, we did not find significant direct effects from stay-at-home orders on physical and mental well-being. Well-being may not be affected by stay-at-home orders *per se* but, possibly, only when individuals’ functional well-being is negatively affected and their resilience is reduced, as we found in the current study. A possible alternative explanation for the absence of direct effects on the mental and physical components of well-being is that the threat of stay-at-home orders alone may have affected well-being. Over time, the amount of states that have issued stay-at-home orders has rapidly increased. At one point, 97% of Americans were ordered to stay home (Stracqualursi, 2020, April 12). It could be argued that individuals who live in states without stay-at-home orders in place worry that such orders could be issued in their state at any time, since they are surrounded by states that have already issued such orders. The threat of being restricted by stay-at-home orders and the associated worry or concern, as well as the threat of being infected with COVID-19, may have reduced well-being.

Our results reveal that stay-at-home orders can have a negative indirect effect on all the components of well-being. Individuals who reported lower functional well-being also reported lower resilience—which is, in turn, associated with lower well-being. This pathway is immensely relevant because it indicates that resilience is an antecedent to all the components of well-being, but also that resilience can significantly reduce when people experience lower functional well-being. This finding is in line with research by Wu et al. (2020), who find that resilience predicts well-being. Our findings demonstrate that resilience can be used as a means of attenuating stay-at-home orders’ potential side-effects on well-being. Therefore, we argue that governments should focus their efforts on increasing the resilience of their people. In situations that necessitate restricting movement to protect public health, resilience can be used to support the preservation of people’s well-being.

### Implications for Public Policy

Governmental agencies may pursue several routes in increasing well-being. The first route entails reducing stay-at-home orders’ negative direct effects on social and financial well-being. Governments may reduce stay-at-home orders’ negative effects on financial well-being by addressing both the objective and the subjective financial consequences of these orders. For example, governments may issue programs that protect people’s incomes. However, importantly, the duration and scope of these programs must be made clear in order to reduce financial worries. Alternatively, institutions may maintain social well-being by ensuring that individuals do not lose social support during stay-at-home orders. For example, schools and care homes can foster social well-being through pen-pal projects, in which young children who are unable to attend school exchange letters with elderly people who are lonely due to isolation (New York Times, 2020).

Increasing resilience is the alternative route to maintaining and improving well-being. This route focuses on ensuring a positive impact on the way people cope with adversity. This approach, in turn, may attenuate stay-at-home orders’ negative effects on all the components of well-being. Research has verified that interventions such as psychological education (Dolbier et al., 2010; Chandler et al., 2015), social support (Waaktaar et al., 2004; Abramson et al., 2015), and coping skills (Hechanova et al., 2016) can increase resilience. Regardless of their exact targets, in all cases where such interventions aim to improve resilience, carefully evaluating their effectiveness is immensely important (Chmitorz et al., 2018).

### Limitations and Future Research

Some limitations should be kept in mind when interpreting our findings. Firstly, we do not have access to pre-measures of well-being and resilience scores per state. Therefore, we cannot make statements about absolute levels of well-being or how stay-at-home orders have affected them. Our data does not show how resilience and well-being evolve over time, so it is possible that these effects were transient and short-lived. In addition, it should be taken into account that the representativeness of our sample, gathered via an online panel provider, may be limited. For example, there was an overrepresentation of participants from California, and an overrepresentation of females in our sample. Another downside of our natural experiment is the *a priori* differences between the states we selected. In our results, we controlled for differences in age, gender, income, and the number of new coronavirus infections, but the possibility remains that we omitted some variables. For example, we did not measure people’s fear of being infected with the coronavirus, or the number of essential workers in states with or without stay-at-home orders. Also, there is increasing evidence that the pandemic affects ethnic groups differently, which we cannot capture or control for with our data. Furthermore, our data only measured whether individuals were in states with stay-at-home orders, and not whether people worried that their states would soon enforce stay-at-home orders. Therefore, with the current data, it is impossible to determine whether worries of impending stay-at-home orders affected well-being in states without stay-at-home orders similarly to how actual stay-at-home orders affected well-being in states where such orders were already in place. In addition, we did not measure whether individuals in states without stay-at-home orders restricted their movements voluntarily. However, we believe this does not limit the interpretation of our results, as other research shows that mobility was lower in states with stay-at-home orders than those without (Engle et al., 2020). Our adapted measure of functional well-being passes thresholds for reliability, but the psychometric properties should be improved and verified by future research. Finally, our data’s cross-sectional nature limits our ability to draw causal inferences. We argue that low functional well-being can reduce resilience, but this finding should be replicated with longitudinal data.

Based on our current findings and the limitations we mention above, we can make several recommendations for future research. Future studies should employ a longitudinal design to capture the effects on well-being over time and identify positive or negative cycles. It is possible that there is a time lag in the effect of stay-at-home orders on physical and mental well-being. According to Halleröd and Seldén (2013), reduced well-being in one component can create a vicious cycle of negative effects on all the components of well-being. For example, low financial well-being may lead to reduced health (for example, due to stress; Kim et al., 2003)—which may lead to lower social well-being (for example, due to a health-related inability to participate in social activities; Stewart et al., 1989). However, it should be noted that positive cycles may also exist, in which well-being in one component leads to increased well-being in the other components (Halleröd and Seldén, 2013). In addition, future research should address the effect of governmental policies on the effects of stay-at-home orders. This research could elucidate which policies are effective in maintaining well-being. For example, studies could examine whether income protection programs effectively maintain financial well-being and whether distinct social-distancing policies have distinct effects on social well-being. Another relevant avenue for future research is the impact that restrictions have on the execution and implementations of coping strategies. It could be studied whether the impact of restrictions on well-being is less for individuals with coping strategies that remain available or possible during stay-at-home orders (e.g., individual sports) versus individuals whose coping strategies are no longer possible (e.g., team sports). In addition, it could be studied whether the impact of stay-at-home orders on well-being differs for essential vs. non-essential workers. Finally, studying how to maintain or improve resilience in the general population after drastic events, such as the outbreak of a pandemic, would also be valuable. This line of research could include testing resilience interventions that could be applied in settings with social distancing in place.

## Conclusion

Government-imposed lockdowns may have side-effects on well-being. We recognize that such lockdowns are essential measures to protect public health and that an uncontrolled pandemic outbreak could create even more substantial adverse consequences for public well-being. However, demonstrating lockdown policies’ potential negative side-effects is important, as is creating strategies to help individuals cope. The results of our study are immensely relevant to crisis situations in which people are ordered to stay home. Our research reveals government-imposed restrictions’ negative effect on the multiple distinct components of well-being. In taking this approach, we contribute to the understanding of consequences of pandemics, and associated governmental restrictions. We show that government restrictions have unique effects on the distinct components of well-being and that focusing on the distinct components of well-being, rather than overall life satisfaction, reveals unique insights. Our finding that resilience mitigates stay-at-home orders’ negative effects on well-being provides policymakers a means to reduce government restrictions’ negative impact. Following our results, governments and policymakers should focus their efforts on strengthening the resilience of their people, which is a key predictor of social, mental, financial, and physical well-being.

## Data Availability Statement

The raw data supporting the conclusions of this article will be made available by the authors, without undue reservation, to any qualified researcher.

## Ethics Statement

Ethical review and approval was not required for the study on human participants in accordance with the local legislation and institutional requirements. Participants provided their electronic written informed consent to participate in this study.

## Author Contributions

AB, JH, and EB were involved in the design of study and the organization of the data collection. AB performed the statistical analyses and drafted the manuscript. All authors discussed the results and contributed to the final manuscript.

## Conflict of Interest

The authors declare that the research was conducted in the absence of any commercial or financial relationships that could be construed as a potential conflict of interest.

## Footnotes

1 This number is calculated by taking the mean of the moving averages from April 06 to April 09 for the statistics of newly confirmed COVID-19 infections provided by Johns Hopkins University, https://coronavirus.jhu.edu/data/new-cases-50-states. We then divided this average by the number of inhabitants per state and multiplied it by 100,000 in order to ensure the numbers’ comparability.
