# Supplementary material for: Coping With Governmental Restrictions: The Relationship Between Stay-at-Home Orders, Resilience, and Functional, Social, Mental, Physical, and Financial Well-Being
Source: Front Psychol. 2021 Jan 13;11:577972. doi: 10.3389/fpsyg.2020.577972 (PMC7838344; doi:10.3389/fpsyg.2020.577972)
Supplement: Supplementary file 1 [file Data_Sheet_1.docx]

**Supplementary materials: Robustness checks**

**1. SEM-analyses with control variables**

We conducted robustness checks data through structural equation modelling. Specifically, we used the Lavaan package in R (Rosseel, 2012) to test whether the direct and indirect effects of stay-at-home orders were robust when controlling for differences in income, age, gender and number of new corona cases per state. The tested model included paths from the control variables (gender, age, income and number of new corona cases in the state) to all dependent variables in the model (well-being dimensions + resilience) (See supplementary figure 1). The exact operationalization of these variables can be found in the method section of the paper. Whereas the model fit is bad (χ2 = 96.22, *p* < .001, *df* = 10; CFI = .926, TLI = .558, RMSEA = .143, SRMR = .054), this model shows that the direct and indirect effects of stay-at-home orders previously reported are robust when these control variables are added to the model (See supplementary table 1).

**Supplementary Figure 1.** Conceptual model of relationship stay-at-home orders, resilience and well-being – including control variables

**
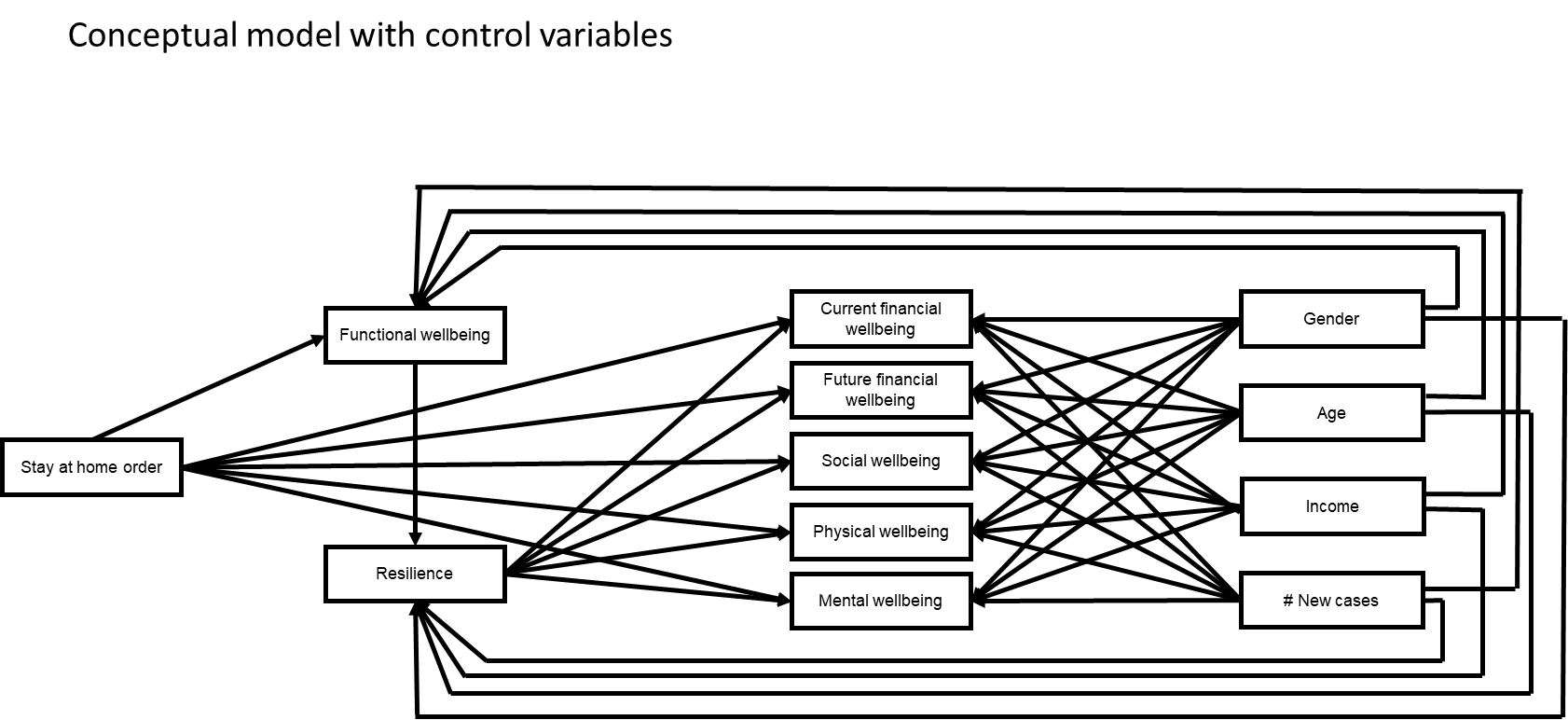
**

**Supplementary Table 1.** Path coefficients for direct and indirect effects of stay-at-home orders on resilience and well-being components controlling for income, gender, age and number of new corona infections

| **Path** | **Coefficient** | **SE (bootstrapped)** | **P-value** | **CI95** |
| --- | --- | --- | --- | --- |
| *Direct effects* |  |  |  |  |
| Stay-at-home order 🡪 Future financial well-being | -0.170 | 0.097 | .080 | -0.360; 0.016 |
| Stay-at-home order 🡪 Current financial well-being | -0.190 | 0.096 | .048* | -0.375; 0.000 |
| Stay-at-home order 🡪 Social well-being | -0.189 | 0.079 | .016* | -0.346; -0.030 |
| Stay-at-home order 🡪 Mental well-being | -0.003 | 0.055 | .957 | -0.116; 0.104 |
| Stay-at-home order 🡪 Physical well-being | -0.011 | 0.081 | .894 | -0.166; 0.150 |
| Stay-at-home order 🡪 Functional well-being | -0.601 | 0.091 | <.001*** | -0.781; -0.427 |
| Functional well-being 🡪 Resilience | 0.128 | 0.041 | .002** | 0.047; 0.208 |
| *Indirect effects* |  |  |  |  |
| Stay-at-home order 🡪 Functional well-being 🡪 Resilience 🡪 Future financial well-being | -0.048 | 0.019 | .010* | -0.092;-0.018 |
| Stay-at-home order 🡪 Functional well-being 🡪 Resilience 🡪 Current financial well-being | -0.035 | 0.014 | .013* | -0.068;-0.013 |
| Stay-at-home order 🡪 Functional well-being 🡪 Resilience 🡪 Social well-being | -0.041 | 0.016 | .010* | -0.080;-0.016 |
| Stay-at-home order 🡪 Functional well-being 🡪 Resilience 🡪 Mental well-being | -0.053 | 0.020 | .009** | -0.100;-0.020 |
| Stay-at-home order 🡪 Functional well-being 🡪 Resilience 🡪 Physical well-being | -0.037 | 0.015 | .011* | -0.072;-0.014 |

* = *p* <.05, ** = *p* <.01, *** = *p* <.001
